# Pelvic Girdle Fluctuating Asymmetry in the Domestic Cat (*Felis catus*): Associations with Age, Sex, and Body Mass

**DOI:** 10.3390/ani16182844

**Published:** 2026-09-09

**Authors:** Piotr Baranowski, Wilhelm Grzesiak

**Affiliations:** 1 Department of Animal Anatomy and Zoology, Faculty of Biotechnology and Animal Sciences, West Pomeranian University of Technology in Szczecin, Klemensa Janickiego 33, 71-270 Szczecin, Poland; 2 Department of Biostatistics, Bioinformatics and Animal Research Methods, Faculty of Biotechnology and Animal Sciences, West Pomeranian University of Technology in Szczecin, Klemensa Janickiego 29, 71-270 Szczecin, Poland; wilhelm.grzesiak@zut.edu.pl

**Keywords:** bilateral variation, developmental instability, morphometric reliability, osteometry, skeletal morphology

## Abstract

Fluctuating asymmetry (FA) is commonly used as a phenotypic indicator related to developmental stability, reflecting variation in an organism’s ability to maintain bilateral correspondence during development. Although pelvic morphology has been extensively investigated in the domestic cat (*Felis catus*), little is known about fluctuating asymmetry of the pelvic girdle and its associations with biological variables such as age, sex, and body mass. This study quantitatively evaluated fluctuating asymmetry of the pelvic girdle in 45 domestic cats using 14 bilateral osteometric traits. Measurement repeatability was assessed using the technical error of measurement (TEM), and traits exhibiting significant directional asymmetry were excluded prior to FA analysis. An individual FA_index was subsequently calculated from the remaining traits and examined in relation to age, sex, and body mass. Six traits exhibited significant directional asymmetry and were therefore excluded from further analyses. Measurement error was low (%TEM: 0.34% to 2.20%) and consistently lower than the corresponding FA values, indicating that the observed asymmetry was greater than the estimated measurement error and was therefore unlikely to be explained primarily by measurement imprecision. The mean FA_index was 0.041 ± 0.032 and showed a positively skewed distribution. Adult cats displayed significantly higher FA_index values than subadults (*p* = 0.007, effect size: r = 0.40), whereas males also exhibited significantly higher FA_index values than females (*p* = 0.009, effect size: r = 0.39). No significant association was detected between FA_index and body mass (R^2^ = 0.014, *p* = 0.432). These findings demonstrate measurable fluctuating asymmetry of the pelvic girdle and indicate that its magnitude varies according to age group and sex within the studied population. The observed difference between subadult and adult cats is consistent with the use of FA as a phenotypic indicator related to developmental stability but does not demonstrate a decline in developmental stability with age. The observed asymmetry may also reflect cumulative functional and biomechanical influences acting on the pelvic skeleton throughout life.

## Simple Summary

Small differences between the left and right sides of the body, known as fluctuating asymmetry, may provide information related to developmental stability and the ability of an organism to maintain similar development of paired structures. This study examined the pelvic bones of domestic cats to determine whether such asymmetry was related to age, sex, and body mass. Adult cats showed greater pelvic asymmetry than subadult individuals, and males showed greater asymmetry than females, whereas body mass was not significantly related to the observed asymmetry. Measurement error was consistently lower than the observed asymmetry, indicating that the detected differences primarily reflected biological variation rather than measurement error. These findings suggest that pelvic fluctuating asymmetry may provide useful information related to developmental stability in domestic cats, while also reflecting biological and functional variation in the feline skeleton.

## 1. Introduction

### 1.1. The Biological Basis of Bilateral Symmetry and Its Developmental Regulation

Bilateral symmetry is a fundamental feature of vertebrate body organization and a defining characteristic of the Bilateria [1,2]. The development of bilaterally corresponding structures on the left and right sides of the body depends on tightly coordinated processes of pattern formation, growth, differentiation, and morphogenesis. These processes are regulated by evolutionarily conserved genetic and molecular networks, including Hox genes and several signalling pathways involved in limb and skeletal development [3,4,5,6,7,8]. Developmental regulation, however, does not operate under completely deterministic conditions. Even within genetically similar organisms developing under comparable environmental conditions, variation may arise from stochastic fluctuations in cellular and molecular processes, as well as from local differences in developmental conditions. Such developmental noise may result in small deviations from the expected correspondence between paired structures despite the overall robustness of developmental regulation [9,10]. Consequently, bilateral symmetry should be regarded not as an absolute developmental outcome, but as the product of a regulatory system capable of buffering minor perturbations while allowing limited random variation to persist. These subtle deviations from perfect bilateral correspondence provide the biological basis for fluctuating asymmetry (FA), which is considered a phenotypic expression of variation in developmental stability [11,12].

### 1.2. Fluctuating Asymmetry (FA) as an Indicator of Developmental Stability

Fluctuating asymmetry (FA) refers to small, random deviations from perfect bilateral symmetry in paired morphological traits and is widely used as a phenotypic indicator of developmental stability [11,12]. Developmental stability describes the ability of an organism to buffer genetic, environmental, and stochastic perturbations during ontogeny and to maintain the expected developmental trajectory and bilateral correspondence of homologous structures [11,13,14]. Within this framework, increased FA may indicate reduced developmental stability when developmental perturbations exceed the capacity of the organism to compensate for them. FA is therefore commonly interpreted as an integrative outcome of multiple factors that may influence developmental processes, including environmental conditions, physiological stress, genetic background, and other sources of developmental variation [13,14,15,16]. Importantly, FA should not be regarded as a specific response to a single stressor or as a direct measure of developmental disturbance. Rather, it represents a phenotypic manifestation of variation in the precision with which bilateral structures develop and may reflect the combined effects of interacting developmental influences throughout ontogeny [15,17]. The interpretation of FA therefore requires consideration of the characteristics of the trait, developmental stage, and potential post-developmental influences that may modify morphological asymmetry [11,13,14].

### 1.3. Skeletal Asymmetry in Mammals: Current State of Knowledge

Skeletal structures provide a particularly informative framework for investigating bilateral asymmetry because their morphology reflects both developmental processes and subsequent functional adaptation. Unlike soft tissues, bones preserve morphological consequences of ontogenetic development while remaining responsive throughout life to mechanical loading, modelling, and remodelling [18]. Consequently, skeletal asymmetry may represent the combined outcome of developmental regulation and later functional influences, and its interpretation requires consideration of both processes. Studies of skeletal asymmetry in mammals have examined a range of anatomical regions, including the skull [19,20], mandible and dentition [21,22], and the appendicular skeleton, particularly the long bones of the limbs [23,24,25]. These investigations indicate that the magnitude and pattern of bilateral asymmetry may vary among anatomical structures and may be associated with environmental conditions, health status, functional demands, and individual developmental history [26]. Within Carnivora, studies of asymmetry have focused predominantly on cranial and masticatory structures, whereas comparatively less attention has been directed toward the postcranial skeleton and pelvic girdle [27,28,29,30,31,32,33]. Research in wild and domesticated mammals has also demonstrated that developmental instability and fluctuating asymmetry may vary among populations and species, emphasizing the importance of interpreting asymmetry in relation to the biological characteristics of the trait and the developmental context [27,31,32]. Although pelvic asymmetry has been documented in carnivorans, comprehensive osteometric studies of pelvic fluctuating asymmetry remain limited, particularly with regard to variation associated with individual biological characteristics.

### 1.4. Functional and Biomechanical Significance of the Pelvic Girdle

The pelvic girdle is a key component of the mammalian musculoskeletal system, linking the axial skeleton with the pelvic limbs and providing attachment sites for muscles involved in locomotion, postural control, and trunk stabilization [34,35]. Its morphology therefore reflects both developmental processes and the functional demands imposed on the skeleton throughout life [36]. The pelvic bones, together with the sacrum and sacroiliac joints, form an integrated biomechanical complex that contributes to the transfer of forces between the vertebral column and the hindlimbs. Locomotor activity subjects the pelvic region to repeated mechanical loading, which may influence bone modelling and remodelling and contribute to individual variation in skeletal morphology. In quadrupedal mammals, pelvic shape has been associated with body size, phylogeny, and locomotor behavior, emphasizing the functional significance of this region within the musculoskeletal system [35]. Evidence from human studies likewise indicates that pelvic morphology and asymmetry may be influenced by mechanical factors, although such findings cannot be directly extrapolated to quadrupedal species [37,38]. The coexistence of developmental and functional influences is particularly relevant to the interpretation of pelvic asymmetry in adult animals, as bilateral differences may reflect both developmental variation and cumulative skeletal adaptation. Accordingly, pelvic fluctuating asymmetry may provide an informative phenotype for investigating variation in developmental stability, while its interpretation requires consideration of potential functional influences acting throughout life.

### 1.5. The Domestic Cat as a Model for Investigating Pelvic Asymmetry

The domestic cat (*Felis catus*) provides a suitable model for investigating pelvic morphology and bilateral asymmetry because of the functional importance of the pelvic region in feline locomotion and the availability of established osteometric and morphometric approaches for assessing feline skeletal variation. Previous studies have documented substantial pelvic variation and sexual dimorphism in domestic cats using conventional linear osteometry and morphometric analyses [39,40,41]. For example, Pitakarnnop et al. [41] evaluated 22 pelvic parameters in 38 domestic cats using digital caliper measurements and identified several pelvic traits that differed significantly between males and females, demonstrating the value of linear osteometry for quantitative assessment of feline pelvic morphology. More recently, geometric morphometric approaches have provided a more comprehensive assessment of pelvic shape and asymmetry. Studies of feline pelvic morphology have used landmark-based methods to characterize variation in pelvic shape, while Altundağ et al. [42] recently applied three-dimensional landmark-based geometric morphometrics within an object-symmetry framework to investigate pelvic directional and fluctuating asymmetry in domestic cats and dogs. Their study explicitly accounted for measurement error through replicate landmark digitization and demonstrated significant components of both directional and fluctuating asymmetry in the examined pelvic landmark configurations. Three-dimensional geometric morphometrics provides important advantages for characterizing overall pelvic shape and complex spatial relationships among anatomical landmarks. Nevertheless, geometric morphometrics and conventional linear osteometry address complementary aspects of skeletal variation rather than representing mutually exclusive approaches. Linear osteometry provides direct and readily interpretable measurements, expressed in metric units, of specific anatomical relationships between homologously defined landmarks. This makes it particularly suitable for explicit comparison of corresponding left and right traits and for quantifying standardized bilateral differences when the research objective is to assess asymmetry in defined anatomical dimensions rather than reconstruct the entire three-dimensional geometry of pelvic shape. The utility of linear osteometry for investigating biologically relevant feline pelvic variation, including sexual dimorphism, has been demonstrated previously [41]. The recent evidence therefore indicates that pelvic asymmetry in domestic cats is no longer an unexplored phenomenon, but important aspects of its biological interpretation remain to be clarified. In particular, the relationship between fluctuating asymmetry assessed from specific bilateral linear dimensions and developmental stability has received less attention than the three-dimensional analysis of overall pelvic shape. The extent to which such osteometric FA varies with age, sex, and body mass also remains insufficiently characterized. These complementary perspectives provide a rationale for investigating pelvic fluctuating asymmetry through repeated bilateral linear osteometry while considering developmental stability together with the potential contribution of lifelong functional adaptation.

### 1.6. Research Gap and Rationale for the Study

Recent research has established that pelvic asymmetry in domestic cats can be characterized using three-dimensional geometric morphometrics, including the assessment of directional and fluctuating asymmetry [42]. However, the relationship between fluctuating asymmetry assessed from specific bilateral osteometric dimensions and developmental stability remains insufficiently characterized. In particular, the extent to which linear pelvic FA varies with age, sex, and body mass has received limited attention. This question is relevant because skeletal asymmetry may reflect developmental variation while also being modified by functional loading and remodelling throughout life, particularly in adult animals. Accordingly, repeated bilateral linear osteometry may complement three-dimensional approaches by providing direct quantitative assessment of asymmetry in defined anatomical dimensions and allowing its relationship with individual biological characteristics to be examined. Such an approach may contribute to a more precise interpretation of pelvic fluctuating asymmetry as a phenotypic indicator related to developmental stability, while acknowledging the potential contribution of lifelong functional influences.

### 1.7. Purpose of the Study

The aim of this study was to quantitatively characterize fluctuating asymmetry of the pelvic girdle in the domestic cat (*Felis catus*) using bilateral osteometric traits and to evaluate its relationship with age, sex, and body mass. We hypothesized that variation in pelvic fluctuating asymmetry may be associated with differences in developmental and functional processes and therefore may vary among individuals with different biological characteristics.

## 2. Materials and Methods

### 2.1. Study Sample

The study sample comprised pelvic girdles from 45 domestic cats (*Felis catus*) of both sexes, selected from an established osteological collection maintained at the Department of Animal Anatomy and Zoology, West Pomeranian University of Technology in Szczecin, Poland. The collection was assembled in the 1970s in connection with anatomical studies on bilateral symmetry and asymmetry of the domestic cat skeleton. The specimens originated from cats obtained at that time from veterinary clinics and an animal carcass disposal facility. Following osteological preparation, complete labeled skeletons were retained in the departmental collection and have subsequently been used for scientific, comparative, and teaching purposes. Historical data associated with the specimens included individual identification number, sex, breed, body mass, and body length. These data were documented in studies conducted on the same animals by Szuba and Nogalski [43] and Szuba [44]. The original skeletal investigations subsequently focused on bilateral characteristics of the skull and mandible, long bones of the limbs, and the atlas. The pelvic girdles included in the present study were not examined in those earlier osteometric analyses. The study sample comprised 20 males and 25 females, with mean body mass of 3.22 ± 0.90 kg and 2.98 ± 0.90 kg, respectively, as derived from the historical records. Pelvic specimens showing evidence of trauma, fractures, neoplastic lesions, congenital abnormalities, or musculoskeletal pathology, including hip dysplasia or degenerative changes, were excluded from the analysis. Age was independently assessed for the purposes of the present study using both cranial and postcranial skeletal criteria. The assessment was based on dental development, including tooth eruption and replacement, and the degree of epiphyseal fusion of the long bones, according to Lutnicki [45] and Habermehl [46], and consistent with the osteological approach previously applied to this anatomical collection by Stacharski et al. [47]. On this basis, the specimens were assigned to two age groups: subadults (6.0–11.5 months; *n* = 14) and adults (>11.5 months; *n* = 31). To minimize potential breed-related variation in pelvic morphology, only cats with documented European Shorthair cat breed status were included in the osteometric study. Consequently, breed did not vary within the analyzed sample and was therefore not included as an independent variable in the statistical analysis.

### 2.2. Osteometric Measurements

**Figure 1:**
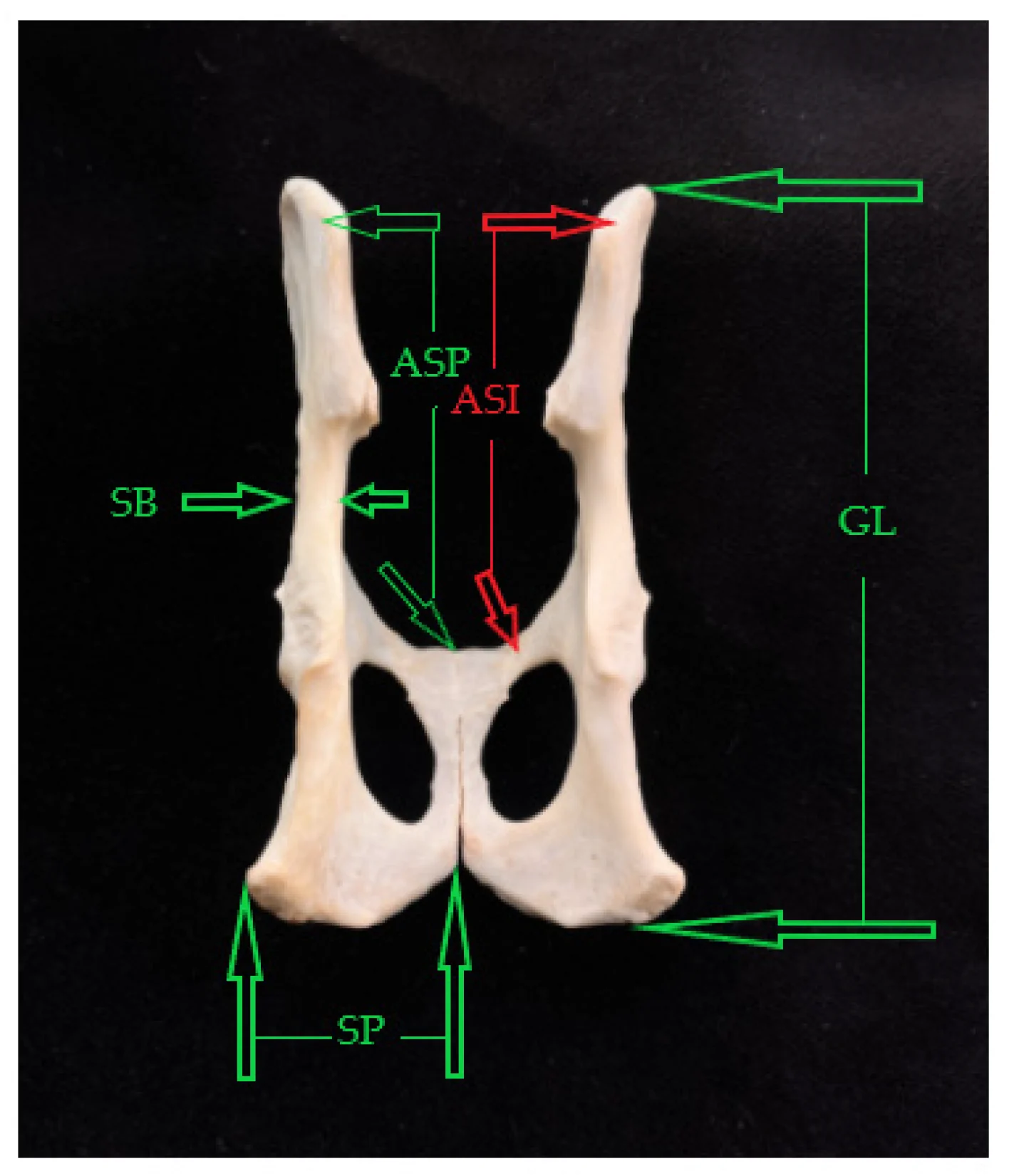
Cat pelvis, dorsal view. Explanation: Measurement landmarks are indicated by arrows. Red indicates traits showing significant directional asymmetry and therefore excluded from the fluctuating asymmetry analysis; green indicates traits retained for the fluctuating asymmetry analysis.

**Figure 2:**
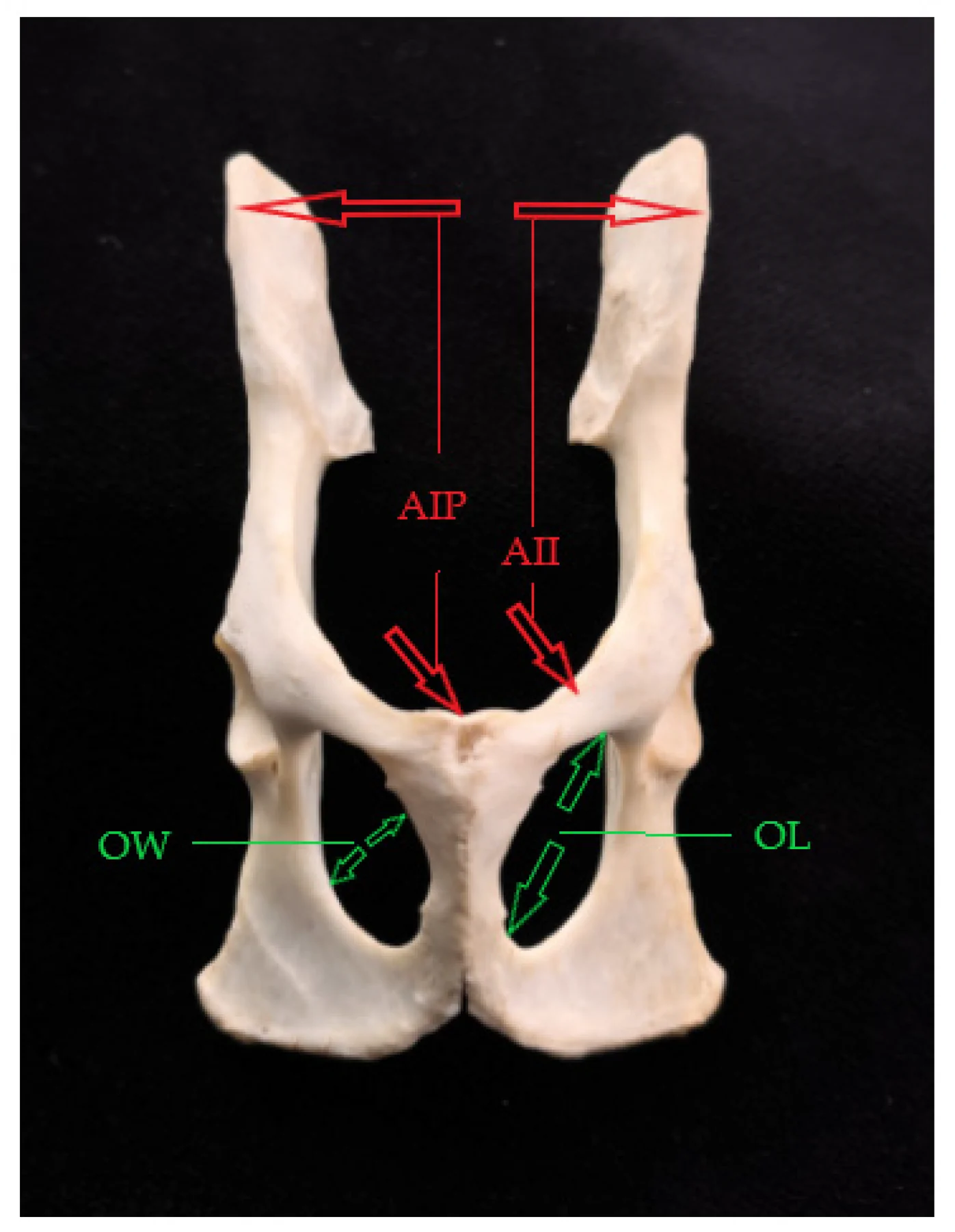
Cat pelvis, ventral view.

**Figure 3:**
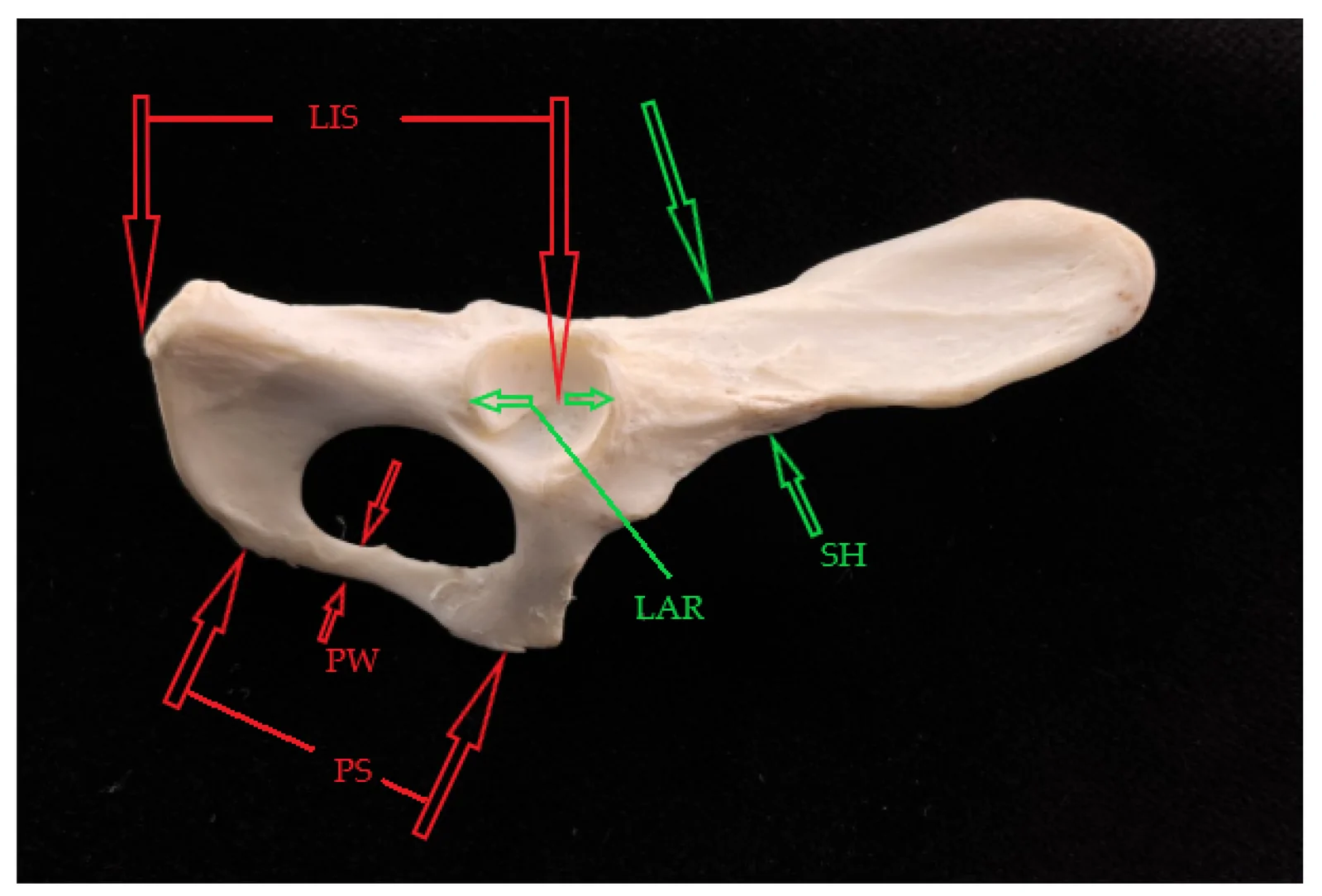
Cat pelvis, lateral view.

Osteometric measurements were obtained using an electronic caliper (ORION 31170-150, HAHN+KOLB Werkzeuge GmbH, Ludwigsburg, Germany) with an accuracy of 0.01 mm. All measurements were taken three times by the same observer at 4- to 6-day intervals to minimize the impact of random observer error and improve the reproducibility of the results. The mean values of the three independent measurements were used for the asymmetry analyses. The selection of traits was based on osteometric standards according to von den Driesch [48] and morphometric studies of the feline pelvis [41]. The full set of osteometric traits considered prior to statistical selection is presented in Table 1, while their anatomical locations are illustrated in Figure 1, Figure 2 and Figure 3.

### 2.3. Assessment of Measurement Error

To assess the precision and repeatability of measurements for each trait, the technical error of measurement (TEM) and the relative error of measurement (%TEM) were calculated. The TEM value was calculated based on the differences between the first and second measurements of each trait, in accordance with the method described by Ulijaszek and Kerr [49]:

$$TEM=\sqrt{\frac{\sum d^{2}}{2N}}$$

where

*d*—the difference between the first and second measurements of the same trait;
*N*—the number of individuals studied.

The relative error of measurement was calculated using the following formula:

$$\%TEM=\frac{TEM}{\overset{ˉ}{x}}\times 100$$

where

$\overset{ˉ}{x}$—mean value of the analyzed trait.

Lower %TEM values were interpreted as indicating higher measurement repeatability.

### 2.4. Analysis of Directional Asymmetry

To identify directional asymmetry (DA) for each analyzed feature, the difference between the values on the left and right sides (L−R) was calculated. The significance of deviations from zero was assessed using the two-sided Wilcoxon signed-rank test for paired samples. Features exhibiting significant directional asymmetry (*p* < 0.05) were considered to violate the assumptions of fluctuating asymmetry analysis and, in accordance with the recommendations of Palmer and Strobeck [50], were excluded from further analysis. The elimination of these features was intended to limit the influence of systematic differences between body sides on the assessment of fluctuating asymmetry.

### 2.5. Analysis of Fluctuating Asymmetry

For each characteristic that did not exhibit directional asymmetry, the fluctuating asymmetry (FA) was calculated using the following formula:

FA = (|L — R|)/((L + R)/2)

where

L—the value of the trait on the left side;
R—the value of the trait on the right side.

The calculations were performed in accordance with the approach proposed by Palmer and Strobeck [50]. Based on the obtained values, an individual fluctuating asymmetry index (FA_index) was determined as the arithmetic mean of the FA values for all traits included in the analysis:

FA_index = (ΣFA_i_)/n

where

FA_i_—the value of fluctuating asymmetry for the i-th trait;
n—the number of traits analyzed.

Only features showing no significant directional asymmetry were used to construct the FA_index. The arithmetic mean of FA values was used as a synthetic indicator of individual fluctuating asymmetry, providing an overall measure of pelvic bone asymmetry for each specimen.

### 2.6. Statistical Analysis

Statistical analyses were performed using Statistica 13 [51]. For the FA_index variable, basic descriptive statistics were calculated, including the mean, standard deviation, minimum and maximum values, and coefficient of variation. The normality of the distribution was verified using the Shapiro–Wilk test.

Due to the non-normality of the FA_index distribution, differences between age groups and sexes were assessed using the Mann–Whitney U test. The effect size was calculated using the formula:

$$r=\frac{Z}{\sqrt{N}}$$

where

Z—denotes the standardized value of the test statistic;
N—denotes the total number of observations.

The relationship between FA_index and body mass was assessed using simple linear regression. The strength of the association was assessed using the correlation coefficient calculated as:

$$r=\sqrt{R^{2}}$$

Statistical significance was accepted at *p* < 0.05 for all analyses.

## 3. Results

### 3.1. Directional Asymmetry

Of the 14 osteometric traits analyzed, six showed significant directional asymmetry and, in accordance with the adopted criteria, were excluded from further analyses of fluctuating asymmetry (Table 2). No significant directional asymmetry was detected for the remaining traits (*p* > 0.05).

### 3.2. Measurement Error

The values of technical measurement error (TEM) and relative measurement error (%TEM) for features that did not exhibit directional asymmetry are presented in Table 3. All %TEM values were low (0.34% to 2.20%), indicating high measurement repeatability. The lowest value was recorded for the GL feature, while the highest was for the SH feature. Measurement repeatability was high for all retained traits.

### 3.3. Fluctuating Asymmetry

A comparison of the mean values of fluctuating asymmetry (FA) and relative measurement error (%TEM) for the traits used to construct the FA_index is presented in Table 4. The highest FA values were recorded for the traits LAR (6.81%), OL (5.46%), and OW (4.93%). For all analyzed traits, FA values consistently exceeded the corresponding %TEM values (Table 4), indicating that the observed asymmetry reflected biological variation rather than measurement error.

**Figure 4:**
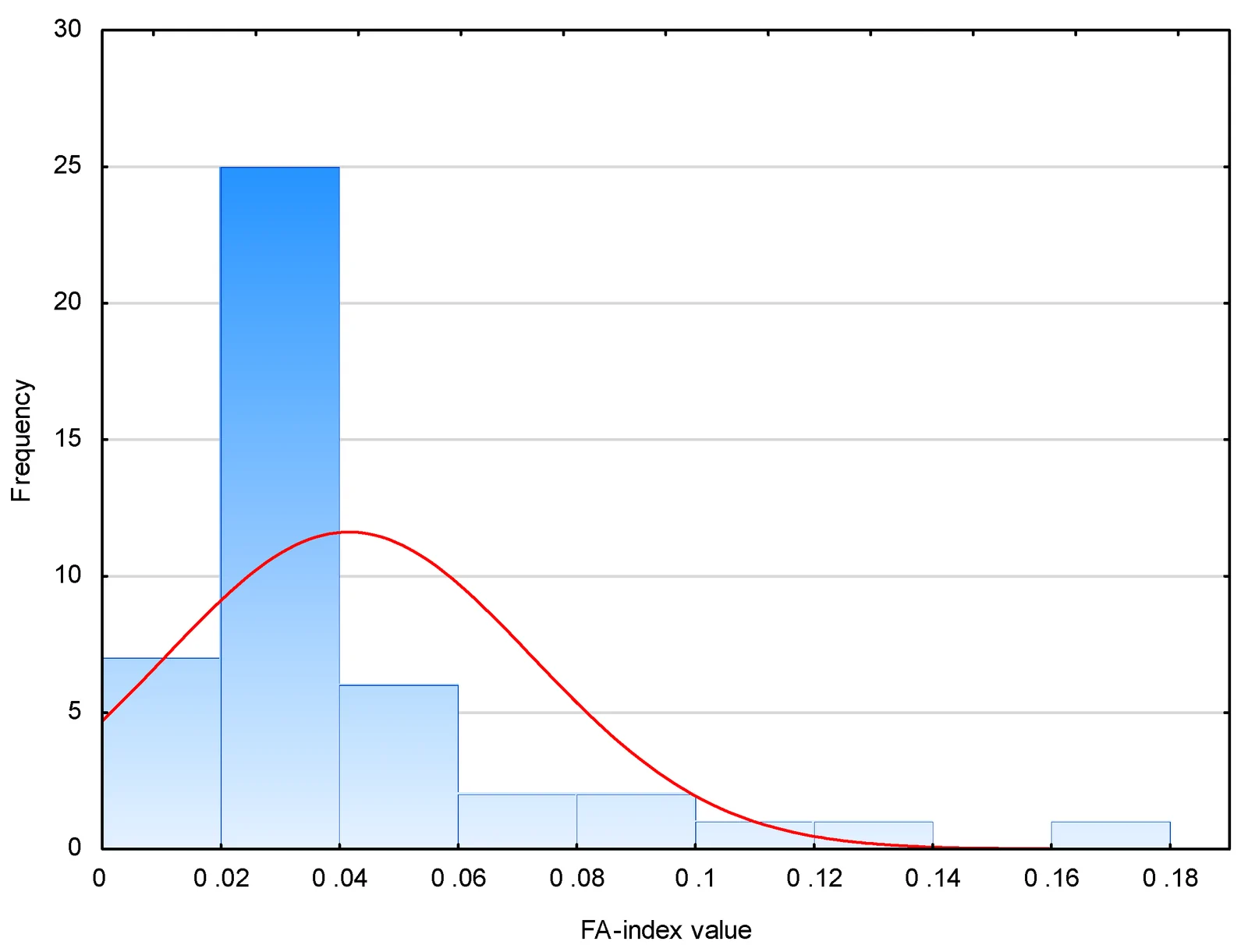
Distribution of the fluctuating asymmetry index (FA_index) values in the study cats. The red line represents the fitted normal distribution.

The mean value of the FA_index was 0.041 (SD = 0.032). The distribution of FA_index values was right-skewed and departed significantly from normality (Shapiro–Wilk: W = 0.7234; *p* < 0.0001) (Figure 4).

### 3.4. Effect of Age and Sex

Adults showed significantly higher FA_index values than subadults (*p* = 0.007, r = 0.40), whereas males had significantly higher values than females (*p* = 0.009, r = 0.39) (Table 5). In both cases, the effect size was moderate (r ≈ 0.40). Adults and males exhibited higher FA.

**Figure 5:**
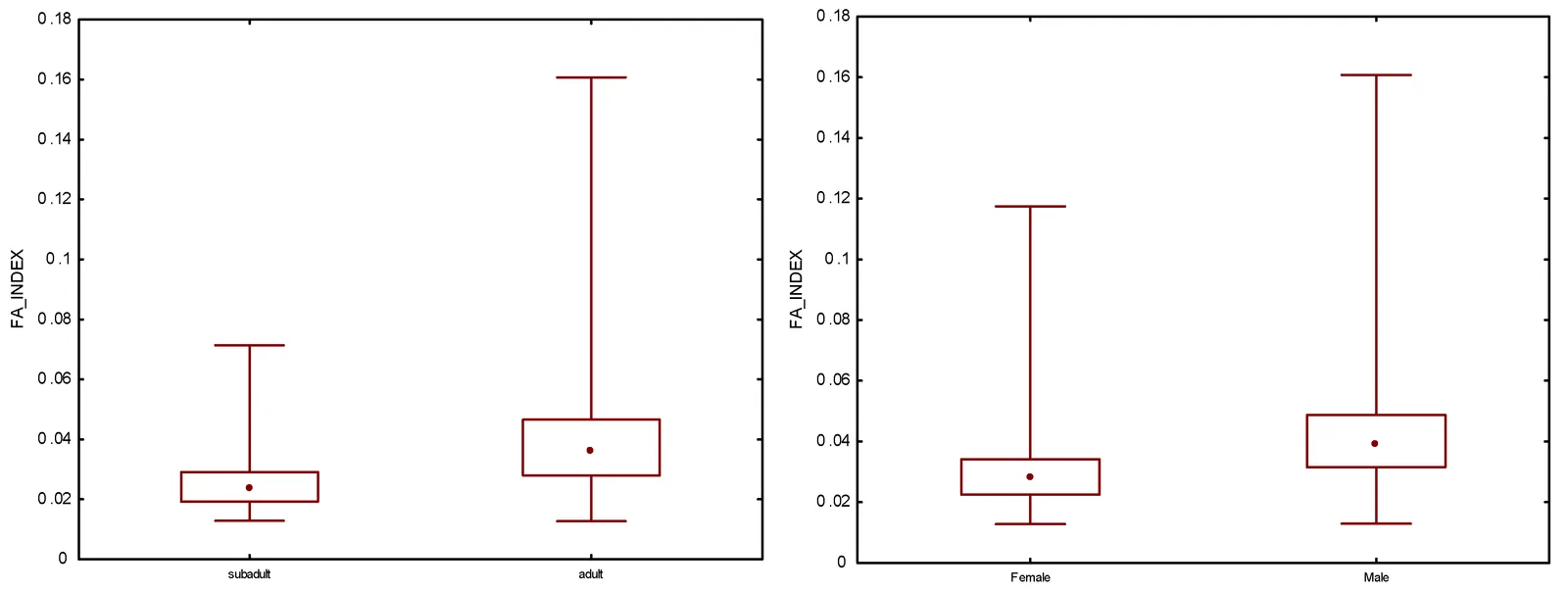
FA_index values depending on age (subadult, adult) and sex (female, male). • Median; 25–75%; Min–Max.

The analysis results are presented in Figure 5.

### 3.5. Relationship with Body Mass

**Figure 6:**
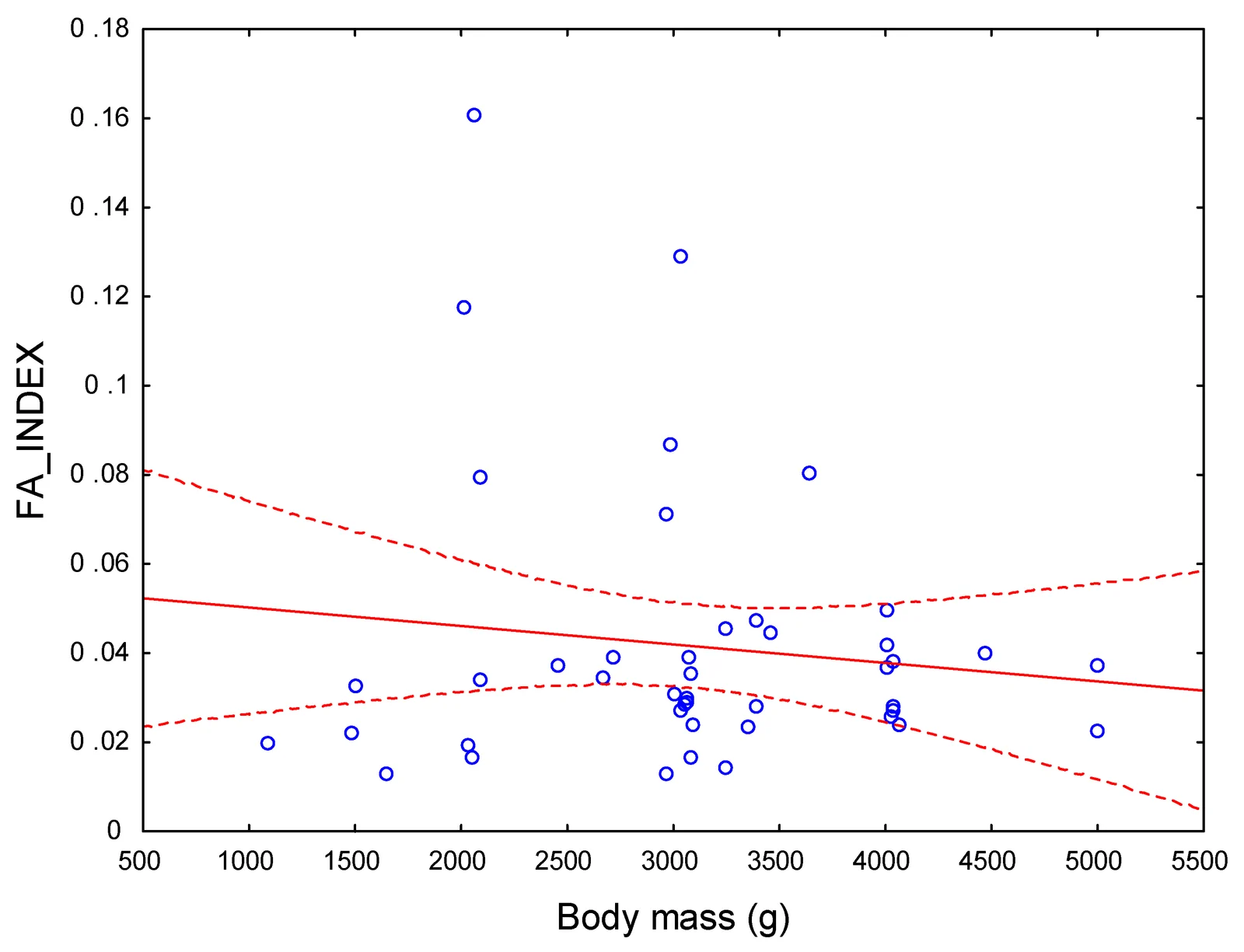
Relationship between the FA_index value and the body mass of the studied cats. The solid line represents the fitted linear regression, and the dashed lines indicate the 95% confidence interval.

Body mass was not significantly associated with FA_index (R^2^ = 0.014, *p* = 0.432). The corresponding Pearson correlation coefficient indicated a very weak relationship (r = 0.12) (Figure 6). Body mass explained only 1.44% of the variance in the FA_index values. No association with FA was detected.

## 4. Discussion

### 4.1. Fluctuating Asymmetry as an Indicator of Developmental Stability

This study suggests that pelvic bone fluctuating asymmetry may provide biologically relevant information related to developmental stability in domestic cats. The mean FA_index value of 0.041 (SD = 0.032) indicates a measurable level of pelvic fluctuating asymmetry within the studied population, which may provide information related to developmental stability. The distribution of the index values was clearly right skewed, which is characteristic of traits related to developmental stability and has been repeatedly described in studies of fluctuating asymmetry [13,50]. Accordingly, most individuals exhibited a low level of asymmetry, whereas only a few showed markedly elevated FA_index values. The higher level of fluctuating asymmetry observed in adults may be associated with skeletal maturation. The effect size for this relationship was moderate (r = 0.40), indicating that age was associated with variability in asymmetry within the population studied. This finding is consistent with the classical view of fluctuating asymmetry as an indicator of developmental stability, reflecting the influence of genetic and environmental factors acting during an organism’s growth [13,50]. The higher FA_index values observed in adults may also reflect the cumulative effects of environmental and functional influences associated with growth and the attainment of skeletal maturity. In skeletal structures, the degree of asymmetry may be further modified by individual differences in biomechanical loading of the pelvic girdle, which plays a key role in locomotion and in transmitting forces between the vertebral column and the hind limbs. However, given the cross-sectional design of the study, the observed difference between subadult and adult cats should not be interpreted as evidence of a progressive increase in fluctuating asymmetry with age. Rather, it may reflect processes associated with skeletal maturation as well as cumulative functional or biomechanical influences.

### 4.2. Effects of Age, Sex and Body Mass on Fluctuating Asymmetry

Significant sex-related differences were also observed. Males exhibited significantly higher FA_index values than females (*p* = 0.009), with an effect size comparable to that observed for age (r = 0.39). This finding indicates that sex was associated with variation in pelvic fluctuating asymmetry within the studied population and may reflect differences between males and females in developmental and functional processes affecting pelvic morphology. The literature emphasizes that males of many species may exhibit greater phenotypic variability and greater sensitivity to environmental factors, which may contribute to higher levels of fluctuating asymmetry [14,52]. In addition, previous studies of geometric morphometry of the feline pelvis have demonstrated marked sexual dimorphism in this structure [39], suggesting that the processes responsible for sexual differentiation may also influence the level of observed asymmetry. The higher FA values observed in males may also result from different growth and maturation trajectories between the two sexes. In contrast to age and sex, no significant correlation was found between fluctuating asymmetry and body mass. This is confirmed by the very small effect size (r = 0.12), indicating that body mass is not a major determinant of pelvic FA. Similar observations have been reported in studies of other skeletal structures, where body mass did not show a clear association with the level of fluctuating asymmetry [53,54]. Similarly, Altundağ et al. [42], in their analysis of pelvic asymmetry in cats and dogs, found no significant association between FA and age, and the effect of body mass was weak or did not reach statistical significance. However, their study excluded animals younger than one year and therefore primarily assessed age-related variation among skeletally mature individuals, whereas our study specifically compared subadult cats (6.0–11.5 months) with adults (>11.5 months). Thus, the difference between the studies may reflect the different age structures of the study populations, with our comparison capturing changes associated with skeletal maturation rather than variation in FA during adulthood. In addition, the two studies used different approaches to assess FA, with Altundağ et al. applying 3D landmark-based geometric morphometrics, whereas the present study used bilateral linear osteometric measurements. Together, these findings indicate that biological factors associated with age and sex may contribute to variation in pelvic fluctuating asymmetry, whereas body mass appears to have a more limited association with FA.

### 4.3. Methodological Aspects of Fluctuating Asymmetry Assessment

The reliability of fluctuating asymmetry assessment was supported by the measurement error analysis because FA represents a subtle biological signal that can easily be obscured by measurement imprecision [50,55]. In this study, %TEM values ranged from 0.34% to 2.20%, indicating high repeatability of the measurements. For all analyzed traits, FA values exceeded their corresponding %TEM values, confirming that the observed level of asymmetry was not solely due to measurement inaccuracies. The greatest difference between FA and %TEM was observed for the LAR, OL, and OW traits, which also exhibited the highest level of asymmetry. Although several individual traits exhibited particularly large differences between FA and %TEM, combining all bilateral traits into a composite FA_index provided a more robust estimate of fluctuating asymmetry as an indicator of developmental instability. An additional methodological strength of the study was the exclusion of traits exhibiting directional asymmetry [55]. This approach is consistent with current recommendations for FA analysis and reduces the risk of attributing directional differences to developmental instability. Directional asymmetry reflects persistent, directional differences between the left and right sides and may represent functional lateralization or adaptive specialization rather than developmental instability. In many cases, DA results from interactions among genetic, developmental, and environmental factors, and its biological significance varies among species and ecological contexts, ranging from functional lateralization and ecological advantages to potential costs associated with injuries [33,56,57,58]. Its exclusion allowed for the separation of the fluctuating asymmetry component from permanent differences between the left and right sides and avoided overestimation of FA values [14,50,55]. Consequently, the resulting FA_index can be interpreted as a phenotypic indicator related to developmental stability rather than as a direct measure of developmental stability. Because developmental instability is expected to affect multiple morphological traits simultaneously, composite indices based on several bilateral characters are considered more informative than single-trait estimates. Accordingly, the use of FA_index in the present study is consistent with current recommendations for multi-trait analyses of fluctuating asymmetry [55,59].

### 4.4. Functional Significance of Pelvic Asymmetry

The pelvis is a biomechanically important structure involved in locomotion and the transfer of loads between the vertebral column and the hind limbs [36,60]. During quadrupedal locomotion, the pelvis also provides attachment sites for muscles involved in propulsion [35]. Consequently, differences in habitual locomotor activity or mechanical loading could potentially contribute to variation in pelvic asymmetry. Its morphology is influenced by both developmental processes and long-term functional forces [61]. Studies conducted on various groups of vertebrates indicate that asymmetry in bone structures may be related to their biomechanical function and the nature of the applied loads [62,63]. In this context, the pelvic asymmetry observed in the present study may reflect not only developmental processes but also the cumulative effects of biomechanical loading throughout life. The results of this study are partially consistent with findings from geometric morphometric studies of the pelvis in cats and dogs [39,42]. Although osteometric and geometric methods describe different aspects of morphological variability, taken together these observations support the view that pelvic morphology reflects the combined influence of developmental history and functional adaptation.

### 4.5. Study Limitations and Future Perspectives

Several limitations of the present study should be acknowledged. First and foremost, the analysis was conducted on a relatively small sample of 45 cats, which limits the generalizability of the results to the broader population of domestic cats. Furthermore, no information was available regarding the environmental history, activity level, or health status of the animals studied. These factors may influence the degree of observed asymmetry and should be taken into account in future studies involving larger populations and a broader range of biological data. Consequently, caution is warranted when generalizing the present findings. Another limitation is the use of classical linear osteometry, which captures selected bilateral pelvic dimensions but does not allow for a comprehensive characterization of three-dimensional shape asymmetry of the entire pelvic girdle. Nevertheless, the TEM and %TEM analyses indicated that the observed level of asymmetry exceeded the measurement error for all analyzed traits, supporting the reliability of the applied osteometric approach. The observed age- and sex-related patterns should therefore be interpreted as biological variation within the studied population, potentially reflecting a combination of developmental processes and cumulative functional or biomechanical influences occurring throughout life. Future studies integrating bilateral linear osteometry with three-dimensional geometric morphometric approaches and detailed biological information may provide a more comprehensive assessment of pelvic asymmetry and its developmental and functional correlates in domestic cats. Three-dimensional approaches may be particularly valuable for capturing aspects of pelvic shape that cannot be represented by individual linear measurements. In a broader comparative context, such approaches may also help relate intraspecific variation in domestic cats to patterns of pelvic morphology observed across Carnivora, where phylogeny and body size have been shown to exert stronger effects on three-dimensional pelvic shape than locomotor behavior [35].

## 5. Conclusions

The present study demonstrates measurable fluctuating asymmetry of the pelvic girdle in the domestic cat. The reliability of the osteometric assessment was supported by measurement error analyses, while the observed associations of fluctuating asymmetry with age and sex, together with the absence of an association with body mass, indicate biologically meaningful variation within the studied population. These findings suggest that pelvic fluctuating asymmetry may provide a useful intraspecific measure of variation in pelvic morphology. However, its interpretation should not be restricted to developmental instability alone, as age-related differences may also reflect cumulative functional and biomechanical influences occurring throughout life. The present study provides a methodological basis for further investigations of pelvic asymmetry in domestic cats. Integrating bilateral asymmetry analyses with three-dimensional geometric morphometric approaches and more detailed biological data may provide a more comprehensive understanding of the developmental and functional factors associated with pelvic morphological variation.

## Figures and Tables

**Table 1:** Osteometric features of the pelvic skeleton of the domestic cat using terminology from the Nomina Anatomica Veterinaria.

Abbreviation	Descriptions of Measurement Abbreviations Illustrated in Figure 1, Figure 2 and Figure 3
GL	The greatest length of the pelvic half (left, right)
SB	The smallest breadth of the shaft of the ilium
SH	The smallest height of the shaft of the ilium
LAR	The greatest length of the acetabulum on the rim
OL	The greatest length of the obturator foramen
OW	The greatest width of the obturator foramen
LIS	The length of the ischium; the distance between the centre of the acetabulum and the lowest part of the ischial tuberosity
ASP	The distance from the cranial dorsal iliac spine to the pubic tubercle
ASI	The distance from the cranial dorsal iliac spine to the iliopubic eminence
AII	The distance between the caudal ventral iliac spine and the iliopubic eminence
AIP	The distance from the caudal ventral iliac spine to the pubic tubercle
PS	The total length of the pelvic symphysis
SP	The distance from the most lateral point of the ischial tuberosity to the medial end of the pubis
PW	Mid-pubis width; the shortest distance between the midpoint of the pelvic symphysis and the obturator foramen

**Table 2:** Analysis of directional asymmetry (DA) for pelvic traits in domestic cats.

Abbreviation	Mean (L–R)	SD	Median (L–R)	Q1	Q3	*p* (Wilcoxon)
GL	−0.23	0.81	−0.09	−0.62	0.24	0.1649
SB	−0.01	0.27	−0.01	−0.17	0.16	0.987
SH	−0.03	0.28	−0.03	−0.20	0.16	0.4411
ASI *	−4.14	3.31	−4.45	−6.60	−1.53	<0.001
ASP	−0.51	3.38	−0.36	−2.12	1.39	0.3635
AII *	−3.57	5.34	−3.20	−7.72	0.29	<0.001
AIP *	−0.67	2.10	−0.46	−1.23	0.24	0.0254
OW	−0.39	1.40	−0.09	−0.37	0.25	0.2473
OL	0.45	2.22	0.10	−0.15	0.30	0.0579
LAR	−0.28	2.80	0.12	−0.38	0.43	0.4131
PS *	−0.89	1.36	−0.56	−1.23	0.07	<0.001
LIS *	0.60	1.46	0.50	−0.27	1.34	0.0084
SP	−0.15	1.28	0.00	−1.13	0.84	0.5556
PW *	0.23	0.50	0.26	0.02	0.53	0.0011

Explanation: L−R, difference between left- and right-side measurements; Q1, first quartile; Q3, third quartile. * indicates significant directional asymmetry (*p* < 0.05); features marked * were excluded from further fluctuating asymmetry analyses.

**Table 3:** TEM and %TEM values for the analyzed osteometric features.

Abbreviation	TEM	%TEM
GL	0.26	0.34
SB	0.15	1.38
SH	0.11	2.20
ASP	0.23	1.21
LAR	0.19	1.71
OW	0.14	1.10
OL	0.18	0.94
SP	0.21	1.11

**Table 4:** Comparison of FA and %TEM values for the traits used to calculate the FA_index.

Abbreviation	FA (%)	%TEM
GL	0.80	0.34
SB	1.98	1.38
SH	4.37	2.20
ASP	4.61	1.21
LAR	6.81	1.71
OW	4.93	1.10
OL	5.46	0.94
SP	4.35	1.11

**Table 5:** Comparison of FA_index according to age and sex.

Variable	Group 1 (n)	Median FA_index (Q1–Q3)	Group 2 (n)	Median FA_index (Q1–Q3)	*p* (Mann–Whitney U)	r
Age	Subadult (14)	0.023 (0.017–0.033)	Adult (31)	0.037 (0.029–0.048)	0.007	0.40
Sex	Female (25)	0.028 (0.022–0.034)	Male (20)	0.039 (0.032–0.049)	0.009	0.39

Explanation: Values are presented as median (Q1–Q3). r indicates the effect size.

## Data Availability

Osteometric measurement data are available from the corresponding author upon reasonable request.
